# Endoscopic necrosectomy is safe in patients with pancreatic walled-off necrosis: Insights from a tertiary center study of 880 procedures

**DOI:** 10.1055/a-2737-6307

**Published:** 2025-11-26

**Authors:** Julie Falkebo Jensen, Joy Stinne Timmner, Amer Hadi, Erik Feldager, August Pilegaard Prahm, Mohamed Ebrahim, Gitte Aabye Olsen, Stine Roug, Srdan Novovic, John Gásdal Karstensen

**Affiliations:** 1 53137 Gastro Unit, Hvidovre Hospital, Hvidovre, Denmark; 2 4321 Department of Clinical Medicine, University of Copenhagen, Copenhagen, Denmark

**Keywords:** Endoscopic ultrasonography, Intervention EUS, Quality and logistical aspects, Performance and complications

## Abstract

**Background and study aims:**

Endoscopic transmural drainage with subsequent endoscopic necrosectomy (EN) has become the first-line treatment for acute necrotizing pancreatitis with walled-off necrosis (WON). There is a growing interest in incorporating EN at the index intervention; however, data about the safety of EN are limited. This case series evaluated the rate and type of adverse events (AEs) associated with EN.

**Patients and methods:**

We retrospectively included consecutive patients with WON from 2012 to 2024 who underwent EN in our tertiary referral center. An AE was defined as any event leading to premature cessation of necrosectomy or requiring intervention either during the procedure or within 24 hours of the procedure.

**Results:**

A total of 235 patients and 880 EN procedures (median: 3, interquartile range: 2–5) were recorded. The median age of patients was 57.5 years, of whom 116 were female (49.3%). Snares were used in most procedures (90.9%), EndoRotor in 4.3%, and both were used in 4.8% of procedures. A total of 14 AEs (1.6%) were identified in 11 different patients (4.7%): 13 bleeds and one pneumoperitoneum. In-hospital mortality was significantly higher in the AE group (45.5%) than in the non-AE group (10.3%, *P* = 0.0004).

**Conclusions:**

AEs are rare in EN but are associated with increased mortality.

## Introduction

Pancreatic walled-off necrosis (WON) is a late complication of acute pancreatitis that is often associated with sepsis, multiorgan failure, and high mortality 1 2 3 . The recommended treatment for WON follows a step-up approach, starting with endoscopic ultrasound (EUS)-guided transmural drainage, usually 4 to 5 weeks after symptom debut 4 5 . In the step-up paradigm, endoscopic necrosectomy (EN) is indicated if endoscopic drainage has proven insufficient. However, there is growing interest in incorporating upfront EN during the index intervention (direct EN, DEN) to reduce the number of reinterventions and shorten the length of stay 6 7 .

Despite the well-established role of EN in management of WON, its safety profile remains poorly described. There is a lack of knowledge about the rate, types, and management of adverse events (AEs) associated with EN, as well as their clinical significance. Polypectomy snares and biopsy forceps are commonly used for EN, but recently, novel devices such as the EndoRotor and over-the-scope grasper have been introduced and shown promising results in improving the efficiency of necrotic tissue removal. However, comparative data on the safety of these devices remain limited 8 .

The primary aim of this study was to evaluate incidence and type of AEs associated with EN in a large, consecutive cohort of WON patients treated endoscopically. As a secondary aim, we sought to investigate management and clinical consequences of AEs.

## Patients and methods

We retrospectively included consecutive patients with WON who were treated with EN in our tertiary referral center between January 2012 and June 2024. Patient age, comorbidities, etiology, procedure details, AEs, and treatment of AEs were extracted from a prospectively maintained database. We defined WON according to the revised Atlanta criteria 1 . Indications for EN included primarily infection, gastric outlet obstruction, and abdominal pain ( Table 1 ). Necrosectomy procedures performed via video-assisted retroperitoneal debridement (VARD), percutaneous metal stent, or transcolonic access were excluded from the analysis.

**Table:** **Table 1** Baseline characteristics of study patients.

	Patients (n = 235)
Age, median, y (IQR)	57.5 (47–69)
Sex, female (%)	116 (49.4)
ASA score, median (IQR)	3 (2–3)
Charlson comorbidity index, median (IQR)	1 (0–2)
Etiology, n (%)	
Gallstones	127 (54)
Alcohol	38 (16.2)
Post-ERCP	31 (13.2)
Idiopathic	26 (11.1)
Other	13 (5.5)
Indication for intervention, n (%)	
Infection	209 (89)
Gastric outlet obstruction	10 (4.2)
Pain	6 (2.5)
More than one of the above	8 (3.4)
Infection and abdominalcompartment syndrome	2 (0.9)
Radiology	
WON diameter CT, median, cm (IQR)	19.2 (16–23)
CT severity index, median (IQR)	7 (5–9)
Modified CT severity index, median (IQR)	8 (6–10)
Total EN procedures, n (%)	
No. of snare procedures	800 (91)
No. of EndoRotor procedures	38 (4.3)
No. of snare + EndoRotor procedures	42 (4.7)
EN procedures, median (IQR)	3 (2–5)
ICU admission n (%)	89 (37.9)
ASA, American Society of Anesthesiologists; CT, computed tomography; EN, endoscopic necrosectomy; ERCP, endoscopic retrograde cholangiopancreatography; ICU, intensive care unit; IQR, interquartile range; WON, walled-off necrosis.	

The primary endpoint was the safety of EN. An AE was defined as any event that led to premature cessation of necrosectomy or that required an unplanned intervention either during the endoscopic procedure or within the first 24 hours after the procedure. AEs were subsequently graded according to the AGREE classification 9 . Pseudoaneurysms and sudden spontaneous bleeding not directly related to a procedure were not categorized as AEs. Secondary endpoints were management of the AEs, clinical resolution, and in-hospital mortality. Clinical resolution was defined as resolution of the WON on computed tomography (CT), with relief of symptoms as well as normalization of C-reactive protein and white blood cell count without the need for antibiotics 10 .

### Procedures

Endoscopic necrosectomy using a snare for debridement of pancreatic walled-off necrosis in a patient, which demonstrates a minimally invasive technique for removing necrotic tissue when drainage alone is insufficient.Video 1

Endoscopic necrosectomy using the EndoRotor system for debridement of pancreatic walled-off necrosis in a patient, which demonstrates a minimally invasive technique for removing necrotic tissue when drainage alone is insufficient.Video 2

The index intervention was a transgastric or transduodenal EUS-guided drainage. Initially, either two 7F double-pigtail stents or one 20-mm lumen-apposing metal stent were inserted to establish access to the WON cavity. EN was subsequently (step-up approach) performed using 10-, 15-, or 25-mm polypectomy snares, EndoRotor 5.0 mm and 3.2 mm (Interscope Inc, Northbridge, Massachusetts, United States), or a combination of both snare and EndoRotor ( Video 1 , Video 2 ).

Nasocystic irrigation catheters were placed in the majority of cases. A number of cases performed with EndoRotor were included in a previously published feasibility study (number of cases: 20) 8 . All EN procedures were performed by four endoscopists with extensive experience in endoscopic management of WON. The procedures were performed under general anesthesia or conscious sedation.

### Statistics

Data are presented as percentages or medians with interquartile range (IQR) for continuous variables. Categorical variables are presented as numbers and percentages. In the analysis of AEs, percentages were calculated relative to the total number of EN procedures performed with snare and EndoRotor. To assess patient data, clinical resolution of WON and clinical outcome percentages were calculated based on number of patients. Mortality rates were compared between patients with and without AEs. The chi-square (χ2) test was used for categorical variables. *P* < 0.05 was considered significant. All statistical analyses were performed using R version 4.2.2 (Posit PBC, Boston, Massachusetts, United States).

## Results

Of 357 WON patients treated during the inclusion period, 235 patients had EN performed. These 235 patients underwent 880 EN procedures (median: 3, IQR: 2–5): 800 were performed with snare (90.9%), 38 with EndoRotor (4.3%), and 42 were performed with both snare and EndoRotor (4.8%). Median age of patients was 57.8 (IQR: 47–69) and 116 patients (49.3%) were female ( Table 1 ).

A total of 14 procedure-related AEs (1.6%) were recorded in 11 different patients (4.7%). Median age of the 11 patients was 62 years (IQR: 55–72.5), three (27.3%) were women and median WON size was 23.2 cm. Median number of EN procedures in these patients was seven (IQR: 3–10). Thirteen of the AEs were bleeding; 12 of these were in patients whose EN used a snare, whereas one patient’s EN used both a snare and EndoRotor. The remaining AE was a pneumoperitoneum and occurred in a patient whose EN used both snare and EndoRotor The majority were classified as AGREE Grade IIIa, reflecting events requiring endoscopic or radiologic intervention. Two events were Grade IIIb, necessitating surgical management, and one was Grade IV, indicating death ( Table 2 ).

**Table:** **Table 2** Characteristics of the 11 patients with AEs.

Patient no.	No. EN during which the AE occurred	AE type	Time from index endoscopy to AE, days	Device involved in AE	Management of AE	AGREE Classification, grade	Time from AE to outcome, days	Clinical outcome	Cause of death
**1**	1	Bleeding	8	Snare	Cessation of procedure CT angiography Embolization of gastroduodenal artery	IIIa	11	Death	MOF
**2**	1	Bleeding	22	Snare	Adrenaline-saline solution Clips	IIIa	22	Death	MOF
**3**	3	Bleeding	13	Snare	Adrenaline-saline solution	IIIa	124	Resolution	N/A
**4**	3	Bleeding	8	Snare	Hemostatic powder Balloon tamponade CT angiography Transfusion	IIIa	29	Resolution	N/A
**5**	4	Bleeding	22	Snare	Cessation of procedure	II	36	Resolution	N/A
**6**	8,9,10	Three bleedings during separate EN	48	Snare	Cessation of procedure CT angiography	II, II and IIIa	31	Resolution	N/A
**7**	8	Bleeding	22	Snare	Adrenaline-saline solution Hemostatic powder Clips CT angiography Transfusion	IIIa	70	Resolution	N/A
**8**	12,14	Two bleedings during separate EN	98	Snare	Adrenaline-saline solution Clips Cessation of procedure	II and IIIa	31	Death	MOF and spontaneous bleeding
**9**	6	Bleeding	39	Snare	Adrenaline saline solution Transfusion CT angiography Re-gastroscopy Balloon tamponade Explorative laparotomy	IIIb	50	Death	MOF
**10**	2	Bleeding	12	Snare + EndoRotor	Adrenaline-saline solution Clips Limited APC Balloon tamponade CT angiography Re-gastroscopy	V	1	Death	Bleeding
**11**	3	Pneumo-peritoneum	24	Snare + EndoRotor	Ultrasound-guided drainage Desufflation Laparoscopic peritoneal lavage	IIIb	74	Resolution	N/A
AE, adverse event; APC, argon plasma coagulation; CT, computed tomography; EN, endoscopic necrosectomy; MOF, multi organ failure; N/A, not available.									

### Bleeding-related AEs

Clinically significant intraprocedural bleeding during endoscopic necrosectomy. Hemospray failed; balloon tamponade achieved hemostasis. CT angiography confirmed no active extravasation and the patient experienced no re-bleeding.Video 3

Three bleeding episodes were successfully managed with adrenaline-saline solution, clips, or both. Six bleeding episodes led to cessation of the EN, mostly due to impaired visualization during the procedure or because even minimal contact with the mucosa or granulation tissue triggered bleeding. In two of these six bleeding episodes, CT angiography was performed; one CT angiograph revealed bleeding from the gastroduodenal artery, which was successfully managed with embolization, whereas the other showed no evidence of extravasation. In the remaining four of these six bleeding episodes, only transient bleeding occurred, and no signs of re-bleeding were observed in any of the six bleeding episodes.

In two other bleeding episodes, hemostasis was attempted with adrenaline-saline solution and hemostatic powder (Hemospray (Cook Medical, Winston-Salem, North Carolina, United States) or Nextpowder (NEXT BIOMEDICAL, Incheon, South Korea). Further, one of these episodes was treated with endoscopic clips and the other with balloon tamponade ( Video 3 ). Blood transfusion was administered in both bleeding episodes and the patients remained stable. Both patients were immediately transferred for CT angiography, which showed no extravasation. There were no signs of re-bleeding.

One bleeding episode was managed with adrenaline-saline solution, argon plasma coagulation, clips, and balloon tamponade. The patient was immediately transferred for CT angiography, which showed no extravasation. After the procedure, a transfusion was administered due to a drop in hemoglobin from 6.5 to 5.8 g/dL in 1 hour. Several hours after the EN, blood was aspirated from the nasogastric tube and a new CT angiograph was performed, revealing extravasation in the fundus of the stomach. The patient subsequently suffered a cardiac arrest in the intensive care unit (ICU) but regained spontaneous circulation. Emergency esophagogastroduodenoscopy (EGD) was performed; however, the bleeding could not be controlled due to poor visualization, and the patient passed away later that evening.

Finally, one bleeding episode was managed with injection of adrenaline-saline solution and blood transfusion. Despite an estimated blood loss of 500 to700 mL, the patient remained stable. The following days, the patient developed abdominal pain, blood was aspirated in the gastric tube, and there was a drop in hemoglobin from 5.1 to 4.0 g/dL. CT angiography was performed due to suspected re-bleeding but showed no extravasation. The CT was followed by an EGD, which showed bleeding of more than 2 L. The bleeding was temporarily controlled using hemostatic powder. Two hours later, a new bleeding occurred of 500 mL in the gastric tube. The patient was transferred to the ICU. The EGD was repeated, with additional evacuation of 3 L of blood, injection of adrenaline-saline solution, and removal of the endoprosthesis. Closure of the stoma was attempted using a clip; however, this proved unfeasible. Instead, a balloon tamponade was applied and the patient was taken to the operating room for an exploratory laparotomy, during which the stoma was successfully closed, thus compressing the cavity and achieving hemostasis.

### Non-bleeding AEs

One patient developed symptoms of pneumoperitoneum and peritonitis 13 hours after an EN procedure (performed using CO₂ insufflation). A CT scan of the abdomen showed no evidence of gastrointestinal perforation. Based on the scan and clinical presentation, it was suspected that air and fluid had leaked from the site of necrosectomy into the peritoneal cavity. Ultrasound-guided percutaneous drainage was performed, yielding brownish fluid, which was presumed to originate from the WON. The pneumoperitoneum was subsequently managed with desufflation and laparoscopic peritoneal lavage. The patient recovered fully and resolved clinically.

### Clinical outcomes of patients with AEs

Among the 11 patients who experienced AEs, clinical resolution was observed in six patients (55%), whereas five patients (45.5%) died during hospitalization before resolution of their WON. Median time from index intervention to first AE was 22 days. In-hospital mortality rate was significantly higher in the AE group (45.5%) than in the group without AEs (10.3%, *P* = 0.0004). In comparison, non-AE patients who also underwent six to eight EN procedures (n = 42) had a median age of 58 years, 40.5% were female, and a median WON size of 20.7 cm, with a mortality rate of 16.7%. The average hospital stay from the first AE to death in these five patients was 23 days. The average hospital stay from the first AE to resolution in the six patients who survived was 61 days.

## Discussion

In this tertiary center study of 880 EN procedures, we found EN to be safe, with an AE rate of 1.6%. Most AEs were bleeding-related (92.9%). The in-hospital mortality rate was significantly higher in the AE group (45.5%) than in the group without AEs (10.3%), underscoring that AEs themselves are likely the main driver of poor prognosis rather than the number of procedures and baseline patient characteristics

**Fig. 1:**
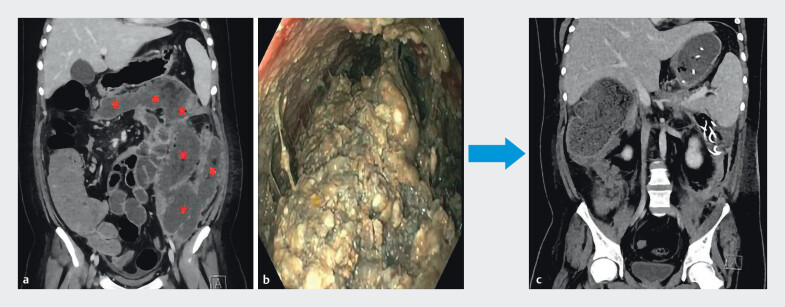
Walled-off necrosis (WON) in a young male patient before and after endoscopic necrosectomy (EN) with snare. **a** Computed tomography (CT) scan showing WON (red stars) before EN with snare. **b** endoscopic view of WON during EN with snare. **c** CT scan showing resolution of WON after nine EN procedures performed over 5 weeks with snare.

Current European Society of Gastrointestinal Endoscopy guidelines recommend EN as part of a step-up approach if the patient does not improve with drainage. However, it has recently been demonstrated that DEN is an efficacious and safe procedure that shortens length of hospital stay 6 7 . Our data support the fact that EN is safe, thus challenging the current step-up approach. Choice of device, such as snare versus EndoRotor, may influence efficacy of EN. However, it is difficult to compare different EN devices due to absence of a validated quality assessment for EN. In addition, characteristics of necrosis may vary, with the snare often proving more effective in some cases, whereas in others, the EndoRotor may be preferable ( Fig. 1 ). Further research comparing different types of EN and identifying the most effective and safest strategies for managing AEs is surely warranted.

**Fig. 2:**
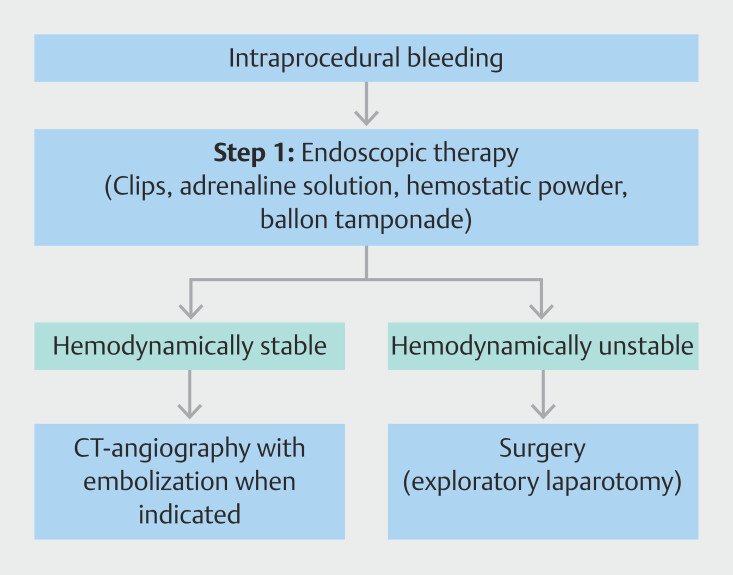
Management algorithm for intraprocedural bleeding during endoscopic necrosectomy. Stepwise approach including initial endoscopic hemostasis (adrenaline–saline solution, clips, hemostatic powder, balloon tamponade), followed by CT angiography with embolization if the patient is stable, and surgical exploration in unstable patients or if prior management fail.

Managing AEs can entail multiple strategies that include adrenaline-saline solution, clips, embolization, hemostatic powder, balloon tamponade, and exploratory laparotomy, which emphasizes the need for a multidisciplinary approach to treatment of these patients ( Fig. 2 ). Further studies specifically designed to characterize the anatomical origin of the bleeding are warranted because that may help refine management strategies and improve outcomes. More consistent and reliable methods for managing AEs are also needed because AEs significantly impact clinical resolution and patient outcomes.

The strengths of this study include its large cohort and inclusion of consecutive patients. However, limitations include its retrospective design, single-center data, and the 12-year inclusion period with potential heterogeneity from changes in technology, techniques, and personnel over time. Recall bias and underreporting of AEs are possible, although all patients were hospitalized, monitored daily with biochemical testing and imaging, and data were recorded in a prospectively maintained database, which reduces this risk. In addition, all patients received prophylactic low-molecular-weight heparin prior to EN, which may have mitigated anticoagulation-associated risk of bleeding and should be considered when interpreting our findings. Spontaneous bleeding events such as those caused by pseudoaneurysms were intentionally excluded because the study specifically aimed to evaluate procedure safety. Furthermore, all EN procedures were performed by four endoscopists with extensive experience in endoscopic management of WON, which might limit generalizability of our findings.

## Conclusions

In conclusion, EN is associated with a low risk of AEs. However, once they occur, AEs are associated with increased mortality.

## References

[1] Banks PA Bollen TL Dervenis C Acute Pancreatitis Classification Working Group. Classification of acute pancreatitis--2012: revision of the Atlanta classification and definitions by international consensus Gut 2013 62 102 111 23100216 10.1136/gutjnl-2012-302779

[2] Ebrahim M Werge MP Novovic S Prediction of admission to intensive care unit and 1-year mortality after acute pancreatitis with walled-off pancreatic necrosis: A retrospective, single-center cohort study Pancreas 2024 53 e386 e394 38416852 10.1097/MPA.0000000000002314

[3] Schepers NJ Bakker OJ Besselink MG Dutch Pancreatitis Study Group. Impact of characteristics of organ failure and infected necrosis on mortality in necrotising pancreatitis Gut 2019 68 1044 1051 29950344 10.1136/gutjnl-2017-314657

[4] Arvanitakis M Dumonceau JM Albert J Endoscopic management of acute necrotizing pancreatitis: European Society of Gastrointestinal Endoscopy (ESGE) evidence-based multidisciplinary guidelines Endoscopy 2018 50 524 546 10.1055/a-0588-5365 29631305

[5] Working Group IAP/APA Acute Pancreatitis Guidelines IAP/APA evidence-based guidelines for the management of acute pancreatitis Pancreatology 2013 13 e1 15 24054878 10.1016/j.pan.2013.07.063

[6] Olsen GA Schmidt PN Hadi A Accelerated vs step-up endoscopic treatment for pancreatic walled-off necrosis: A randomized controlled trial (ACCELERATE). Clin Gastroenterol Hepatol 2025:S1542–3565(25)00701–3 10.1016/j.cgh.2025.08.007 40835042

[7] Bang JY Lakhtakia S Thakkar S United States Pancreatic Disease Study Group. Upfront endoscopic necrosectomy or step-up endoscopic approach for infected necrotising pancreatitis (DESTIN): a single-blinded, multicentre, randomised trial Lancet Gastroenterol Hepatol 2024 9 22 33 10.1016/S2468-1253(23)00331-X 37980922

[8] Olsen GA Schmidt PN Novovic S Novel powered 5.0-mm endoscopic debridement catheter for endoscopic transmural necrosectomy of pancreatic walled-off necrosis: a case series of consecutive patients from a tertiary referral center (with video) Gastrointest Endosc 2024 99 267 270 37865281 10.1016/j.gie.2023.10.044

[9] Nass KJ Zwager LW van der Vlugt M Novel classification for adverse events in GI endoscopy: the AGREE classification Gastrointest Endosc 2022 95 1078 1.085E11 34890695 10.1016/j.gie.2021.11.038

[10] Karstensen JG Novovic S Hansen EF EUS-guided drainage of large walled-off pancreatic necroses using plastic versus lumen-apposing metal stents: A single-centre randomised controlled trial Gut 2022 72 1167 1173 10.1136/gutjnl-2022-328225 36446550

